# Effects of Green Brazilian Propolis Alcohol Extract on Nociceptive Pain Models in Rats

**DOI:** 10.3390/plants9091102

**Published:** 2020-08-27

**Authors:** Mohammed T. Al-Hariri, Turki S. Abualait

**Affiliations:** 1 Department of Physiology, College of Medicine, Imam Abdulrahman Bin Faisal University, P.O. Box 2114, Dammam 31541, Saudi Arabia; 2 Department of Physical Therapy, College of Applied Medical Sciences, Imam Abdulrahman Bin Faisal University, Dammam 34212, Saudi Arabia; tsabualait@iau.edu.sa

**Keywords:** propolis, rats, antinociceptive pain, acetic acid, formalin test

## Abstract

Pain is one of the most common symptoms encountered in the medical practice. None of the management procedures used currently offer a complete relief for patients suffering from nociceptive pain. New treatment strategies for pain management are needed. Propolis has been used in traditional medicine to relieve various types of pain. The aim of the current study was to investigate the potential effects of the green Brazilian propolis alcohol extract in vivo on the nociceptive and inflammatory pain models in rats. Rats were distributed into three random groups (*n* = 6); Group I: control group received normal saline intraperitoneally (i.p.); Group II: treated with green Brazilian propolis alcohol extract (P50 mg/kg i.p.); Group III: treated with P100 mg/kg i.p. After sixty minutes, 50 μL of 5% formalin was injected subcutaneously into the dorsal surface of the right hind paw. The nociceptive response was identified by counting the number of flinches of the injected paw. The number of flinches was counted for the period of 0–5 min (early phase; neurogenic) and 10–60 min (late phase; inflammatory). Thermal hyperalgesia was assessed using three-paw withdrawal latency measurement with ten minutes intervals using a planter analgesic meter. Abdominal writhe (contraction) was induced by i.p. injection of acetic acid (1 mL of 2%). The results showed that green Brazilian propolis alcohol extract caused a significant inhibition of acetic acid-induced pain and significantly increased the pain threshold against infrared and formalin tests. The promising antinociceptive and anti-inflammatory properties of propolis and/or its active constituents as natural compounds in the present study indicates that it merits further studies in pain.

## 1. Introduction

Pain is an uncomfortable feeling that involves motivational, cognitive and affective aspects, and sometimes act as an alarm to minimize the contact with harmful agents [1]. Pain is initiated by nociceptors (specialized peripheral sensory neurons) that are activated by noxious stimuli (i.e., chemical, mechanical and thermal stimuli) due to tissue damage and/or injury. These nociceptors are usually found in the muscle, cutaneous tissues, connective tissues, vessels, viscera and bone [2]. The treatment options include administration of analgesic and anti-inflammatory agents [3]. However, when pain persists, it may affect the quality of life of a person who suffers from it [4].

Natural products have played an essential role in the discovery of analgesic drugs [5]. In the last three decades the interest in natural products has greatly increased which has been led by the World Health Organization (WHO). The WHO recently published a document entitled “WHO traditional medicine strategy: 2014–2023”, in which it endorsed and supported the development and implementation of action plans to strengthen the role traditional medicines in public health [6].

Due to frequent adverse effects associated with pain therapy, the search for new and effective drugs with either no or least undesirable effects is necessary [7] Different chemical compounds such as flavonoids having potent analgesic effects have been obtained from natural sources, resulting in novel lead compound classes for designing of analgesic drugs [8]. Moreover, cinnamic acid, phenolic acids and caffeic acid, demonstrated to have anti-inflammatory and antinociceptive effects are considered promising phytochemicals that show distinct physiological and pharmacological properties [2,9].

Propolis (bee glue) is a natural resinous mixture produced by honey bees by combining bee-released compounds with exudate gathered from various plant sources [10].

Several studies conducted with propolis have identified more than 500 constituents to date—most notably are caffeic acid, phenolic acid, terpenes, flavonoids, cinnamic acid as well as 64 esters [11,12].

Propolis presents many physiological and pharmacological properties such as antimicrobial (antiviral, antibacterial, antiparasitic and antifungal) [13,14], immunomodulatory [15], anti-inflammatory [16] antioxidant [14], and, antitumor activities [17].

Interestingly, a clinical study confirmed that administration of propolis active compounds could be a better adjuvant therapy in patients with human cancer [11].

A recent study reported that propolis has beneficial effects on reducing post prandial blood glucose, inflammatory cytokines, insulin resistance and serum insulin. Furthermore, the study found that propolis can also prevent liver and renal dysfunction in patients with Type 2 diabetes mellitus [18].

Currently, propolis and its extracts are used in different pharmaceutical dosage forms such as creams, powder, throat lozenges, capsules, mouthwash solutions and soaps. Current medical applications of propolis include dermatological preparations which are useful in treatment of burns, herpes simplex, neurodermatitis, genitalis, wound healing, and acne. It is also used in formulations for cold syndrome (common cold, flu-like infections and upper respiratory tract infections). Propolis is also used in toothpastes and mouthwashes to treat stomatitis and gingivitis and to prevent dental caries [19].

Propolis ethanolic (of Bulgarian [20], Irani [21] and Chinese [22]), hydroalcoholic (of red Brazilian propolis [23]) and water (of Iraqi [24] and Moroccan Black [25]) extracts have shown considerable in vivo anti-inflammatory and antinociceptive activities in animal models. The available data varies based on the chemical composition of propolis, plant sources, and, collecting season [20,23,26,27].

There are limited data on the antinociceptive effect of green Brazilian propolis alcohol extract. Therefore, the present study aimed to evaluate the antinociceptive effect of alcohol extracts of green Brazilian propolis on models of nociceptive and inflammatory pain in rats.

## 2. Methodology

### 2.1. Experimental Animals

Wistar male albino rats (250–300 g) were obtained from the animal house of the Imam Abdulrahman Bin Faisal University. Animals were maintained with free access to water and food at controlled room “temperature 25–29 °C, 12-h light and 12 h darkness cycles” [28]. The experiment was performed after approval of the protocols by University Institutional Review Board Committee (lRB 2015-03-114) and was carried out in accordance with the current guidelines for the care of laboratory animals. Rats were distributed into three random groups (*n* = 6); Group I: control group that received normal saline intraperitoneally (i.p.); Group II: treated with high flavonoids green Brazilian propolis (55%) alcohol extracts (Uniflora) 50 mg/kg (P50) i.p. and Group III: treated with 100 mg/kg (P100) i.p. Animal groups were treated 60 min, before the test [29]. These doses were selected based on previous significant biologic findings of propolis extracts [30,31,32].

### 2.2. Animal Models of Pain

#### 2.2.1. Thermal Hyperalgesia (Infrared) Test

Thermal hyperalgesia was assessed using a planter analgesic meter (Ugo Basile, Gemonio, Italy). Rats were placed individually in clear plastic cages and allowed to acclimate for 20 min before testing. The center of a focused beam of infrared heat was applied to the plantar surface of the hind paws. Both timer and bulb were immediately turned off by rat’s paw withdrawal or on reaching a predetermined cut-out (usually 22 s) to prevent tissue damage. The reaction time was recorded when the rats licked their hind paws and jumped at several intervals of 30, 60 and 90 min post propolis administration. The withdrawal latency of hind paws was measured to the nearest 0.1 s [33].

#### 2.2.2. Formalin Test

Rats were treated with different doses (P50 and P100) of propolis i.p. After sixty minutes, 50 μL of 5% formalin was injected subcutaneously into the dorsal surface of the right hind paw using a microsyringe with a 27-gauge needle. Immediately after formalin injection, the animals were placed individually in acrylic observation chambers (320 cm × 40 cm). Mirrors were arranged at angles to allow clear observation of the paws of the animals. Each rat in every group was observed simultaneously from 0 to 60 min following formalin injection. The nociceptive response was identified by counting the number of flinches of the injected paw. The number of flinches was counted for the periods of 0–5 min (neurogenic phase) and 10–60 min (Inflammatory phase) [34].

#### 2.2.3. Abdominal Writhing test

Abdominal writhe (contraction) was induced by i.p. injection of 1 mL of a solution of 2% acetic acid using a 25-gauge injection needle. Five minutes after the administration of acetic acid, the frequencies of abdominal writhing were recorded for sixty minutes and, the latent period, i.e., beginning of the first abdominal writhe after injection, was also observed [9].

### 2.3. Statistical Analysis

Statistical analysis was performed using the Statistical Package for the Social Sciences (IBM SPSS V 21). The normality of data were tested by the Shapiro–Wilk. The number of flinches of all phases of response were calculated for each rat. Data with homogeneity of variance were analyzed using the two-way analysis of variance (ANOVA), and multiple post hoc comparisons were performed using the Tukey’s test. *p*-values < 0.05 were considered to be statistically significant. Data were expressed as mean ± standard error of mean (SEM).

## 3. Results

### 3.1. Inhibitory Effect of Propolis on Thermal Stimuli-Induced Nociception in the Infrared Test

**Figure 1:**
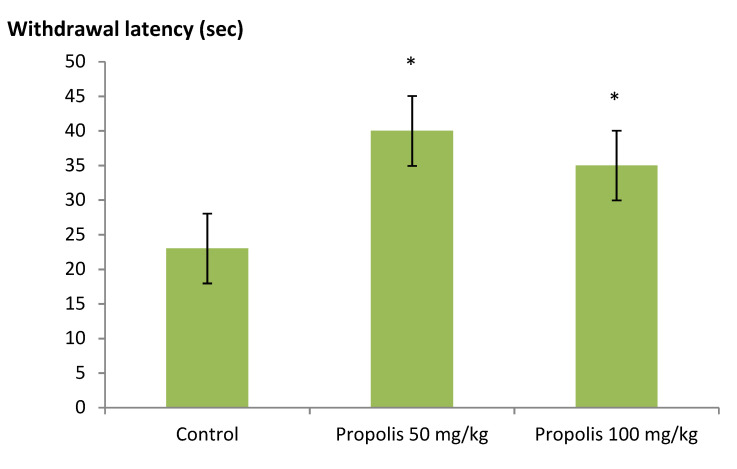
Antinociceptive effect of green Brazilian propolis alcohol extract (P50 and P100) in the infrared test in the rats. The values expressed are mean ± SEM, (*n* = 6).* *p* < 0.05: significantly different for treated (P50 and P100) compared to the control groups as determined by ANOVA analysis followed by multiple comparison post hoc test.

Administration of green Brazilian propolis alcohol extract at doses 50 and 100, mg/kg, i.p. increased the latency time to the nociceptive response in the infrared test. Significant differences (*p* < 0.05) between the mean thermal threshold of P50 and P100 than the control, clearly indicating the analgesic property of the alcohol green Brazilian propolis alcohol extract as presented in Figure 1.

### 3.2. Inhibitory Effects of Propolis on Formalin-Induced Inflammatory Nociception

**Figure 2:**
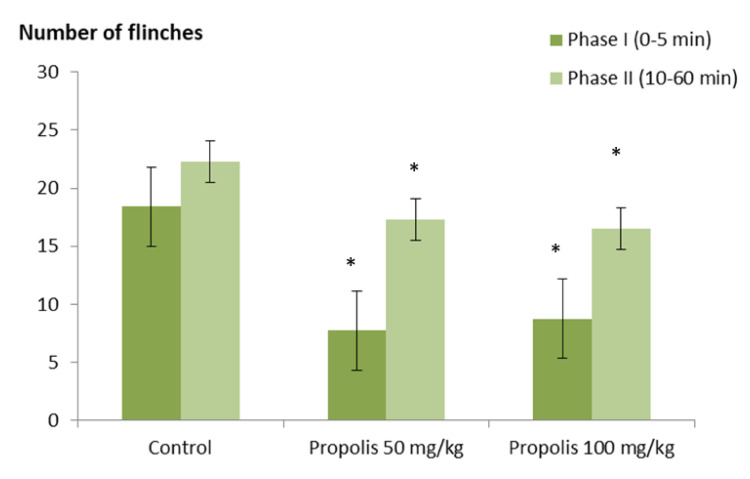
Effect of green Brazilian propolis alcohol extract (P50 and P100) in the formalin-induced nociception model: neurogenic (Phase I) and inflammatory (Phase II). The values expressed are mean ± SEM, (*n* = 6). * *p* < 0.05: significantly different for treated (P50 and P100) compared to the control groups as determined by ANOVA analysis followed by multiple comparison post hoc test.

Figure 2 indicates the effect of the green Brazilian propolis alcohol extract on formalin-induced pain during the acute phase in rats. Both propolis groups (P50 and P100) displayed a significant (*p* < 0.05) reduction in formalin-induced nociceptive behavior monitored by the mean number of flinches across all time points of the neurogenic phase (0–5 min) than the control. Similarly, a significant (*p* < 0.05) pain inhibition in both groups (P50 and P100) was observed in the inflammatory phase (10–60 min) after induction of the noxious stimulus, than the control.

### 3.3. Inhibitory Effect of Propolis on Acetic Acid-Induced Writhing Response

**Figure 3:**
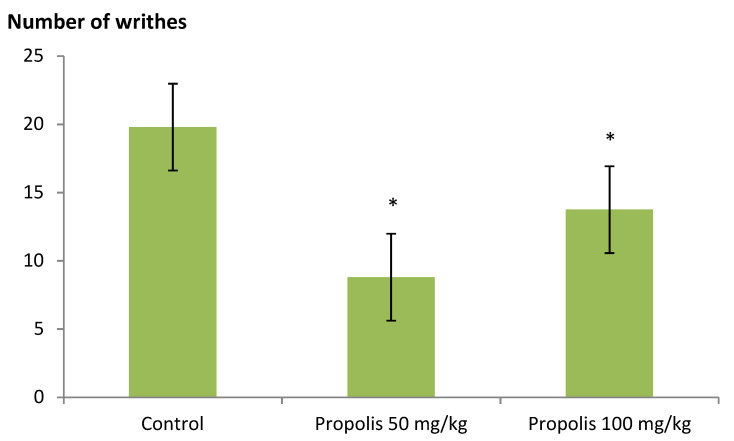
Effect of green Brazilian propolis alcohol extract (P50 and P100) against acetic acid-induced abdominal contraction in rats. The values expressed are mean ± SEM (*n* = 6).* *p* < 0.05: significantly different for treated (P50 and P100) compared to the control groups as determined by ANOVA analysis followed by multiple comparison post hoc test.

The effect of the green Brazilian propolis alcohol extract on the antinociceptive activity via the acetic acid-induced abdominal writhing test is shown in Figure 3. Both propolis doses 50 and 100, mg/kg, i.p. significantly (*p* < 0.05) decreased acetic acid-induced writhing responses than the control group.

## 4. Discussion

This study evaluated the effects of the green Brazilian propolis alcohol extract, using several well-established in vivo nociception assays with different pain stimuli (thermal and chemical) in rats. The results demonstrated that i.p. administration of propolis (P50 and P100) significantly reduced the nociceptive response.

Administration of green Brazilian propolis alcohol extract reduced the number of flinching response evoked by formalin injection during both phases. The early phase started at the time of injection and lasted for about five minutes and the late phase, which started at 10 minutes post-injection and lasted for about 60 minutes. The early phase response results from immediate stimulation of primary afferent fibers (C-fiber activation due to the peripheral stimulus) in the paw that reflects the centrally mediated pain. The late phase response may result from an inflammatory reaction in the peripheral tissue, combined with the activation of wide dynamic range neurons (activation of *N*-methyl-d-aspartate (NMDA) and non-NMDA receptors and NO cascade) in the dorsal horn of the spinal cord [35,36]. This suggests that green Brazilian propolis alcohol extract possesses a biphasic analgesic effect. These findings are in line with other studies that highlighted analgesic effect of propolis. Sun et al. (2018) have shown that different fractions from Chinese propolis extracts enriched in polyphenolic constitutions showed central and peripheral antinociceptive effects in vivo animal models [22]. In another study, Cavendish et al. (2015) had described the antinociceptive effect of Brazilian red propolis hydroalcoholic extract on inflammatory and neurogenic pain, without emotional and motor side effects in rodents [23]. Notably, the main ingredient of propolis (Caffeic acid phenethyl ester), was found to be an effective and safe agent for reducing neuropathic pain by suppressing the P38/NF-κB signal pathway in microglia [37].

Our data showed that green Brazilian propolis alcohol extract caused a significant inhibition of acetic acid-induced pain that reduced the number of abdominal contraction and stretching of hind limbs. The acetic acid-induced nociceptive response may involve two processes; synthesis of inflammatory mediators which can reduce the threshold of nociception and the direct stimulation of the nociceptive afferent fibers due to the reduction of pH [38]. The antinociceptive effect of green Brazilian propolis alcohol extract against acetic acid-induced writhing may occur via local peritoneal receptors or by inhibition of prostaglandin synthesis or action. [39,40]. Another study reported that ethanolic extract of propolis reduced acetic acid-induced abdominal contraction in experimental model [41].

Propolis is a natural product that can be used for the treatment of different gynecological, deontological and, dermatological conditions, in which pain is a common symptom [42]. The inflammatory process involves production and/or release of mediators from neurons or damaged tissues that are responsible for different responses including pain [43,44]. Different chemical mediators such as serotonin, kinins, prostaglandin and excitatory amino acids, are involved in the pathogenesis of inflammation and pain processing responses [43,44].

Thus, the presence of flavonoids in the green Brazilian propolis alcohol may be responsible for these inflammatory mediators [45]. Several studies have tried to explain the mechanisms behind analgesic effect of propolis. One study found that some isolated flavonoid compounds inhibited the gene expression of inflammatory mediators, including IL-13, TNF-α and IL-6 [45]. Moreover, the ability of flavonoids to inhibit the release of arachidonic acid has been documented [24]. Further, flavonoids may be involved in the interactions with GABA-ergic, serotonin and L-arginine–NO system [46].

## 5. Conclusions

The findings of this study are promising in suggesting an important analgesic potential effect of the green Brazilian propolis alcohol extract due to its inhibitory effect on pain threshold. The promising antinociceptive and analgesic properties of propolis and/or its active compound in the present study indicates that it merits further studies in antinociceptive as well as neuropathic pain investigations. Furthermore, it supports the traditional indication of propolis as an alternative natural product in the treatment of several painful and inflammatory associated diseases.

## 6. Limitation

Some limitations to this study should be considered. The antinociceptive effect of propolis was not compared to other pain killer or anti-inflammatory treated groups. In addition, the biphasic effects of i.p. injected propolis on the early and late phases should be further clarified in the future.

## 7. Data Availability

The data used to support the findings of this study are included within the article.
